# Mitochondrial Fusion and ERK Activity Regulate Steroidogenic Acute Regulatory Protein Localization in Mitochondria

**DOI:** 10.1371/journal.pone.0100387

**Published:** 2014-06-19

**Authors:** Alejandra Duarte, Ana Fernanda Castillo, Ernesto J. Podestá, Cecilia Poderoso

**Affiliations:** Institute of Biomedical Investigations (INBIOMED, UBA-CONICET), Department of Biochemistry, School of Medicine, University of Buenos Aires, Buenos Aires, Argentina; Oregon Health & Science University, United States of America

## Abstract

The rate-limiting step in the biosynthesis of steroid hormones, known as the transfer of cholesterol from the outer to the inner mitochondrial membrane, is facilitated by StAR, the Steroidogenic Acute Regulatory protein. We have described that mitochondrial ERK1/2 phosphorylates StAR and that mitochondrial fusion, through the up-regulation of a fusion protein Mitofusin 2, is essential during steroidogenesis. Here, we demonstrate that mitochondrial StAR together with mitochondrial active ERK and PKA are necessary for maximal steroid production. Phosphorylation of StAR by ERK is required for the maintenance of this protein in mitochondria, observed by means of over-expression of a StAR variant lacking the ERK phosphorylation residue. Mitochondrial fusion regulates StAR levels in mitochondria after hormone stimulation. In this study, Mitofusin 2 knockdown and mitochondrial fusion inhibition in MA-10 Leydig cells diminished StAR mRNA levels and concomitantly mitochondrial StAR protein. Together our results unveil the requirement of mitochondrial fusion in the regulation of the localization and mRNA abundance of StAR. We here establish the relevance of mitochondrial phosphorylation events in the correct localization of this key protein to exert its action in specialized cells. These discoveries highlight the importance of mitochondrial fusion and ERK phosphorylation in cholesterol transport by means of directing StAR to the outer mitochondrial membrane to achieve a large number of steroid molecules *per* unit of StAR.

## Introduction

Mitochondria are a key control point for the regulation of steroid hormone biosynthesis. The transport of cholesterol across the intermembrane space from the outer (OMM) to the inner mitochondrial membrane [1] provides the substrate for all steroid hormones. This point is the first and rate-limiting step in steroidogenesis [2], [3], [4] which is facilitated, among other proteins, by the Steroidogenic Acute Regulatory (StAR) protein [5], [6]. StAR protein is synthesized as a 37 kDa preprotein with a typical mitochondrial leader sequence; upon hormonal stimulation it is translocated to yield a mature 30 kDa mitochondrial protein [7], [8]. StAR mediates the rapid flow of cholesterol from the OMM to the IMM, enabling steroidogenic cells to make a large amount of steroids in a short period of time. A small amount of StAR has been reported to elicit cholesterol transport to achieve the maximal rate of steroid synthesis [9]. Compelling previous studies indicate that StAR functionally promotes steroidogenesis exclusively at the OMM, even in the absence of the mitochondrial import sequence and loses activity when it reaches the mitochondrial matrix [10], [11], [12], [13]. Furthermore, it has been reported that StAR’s activity is proportional to how long it remains on the OMM [14]. There is also evidence that inner mitochondrial proteolysis in adrenal cells is essential in cholesterol fluxes, with a role of StAR in the IMM [9], [15]. Thus, the mechanisms of StAR import and processing remain unclear. Once synthesized, StAR preprotein must quickly associate with the mitochondria or otherwise it could be rapidly degraded in the cytosol. The protophonore carbonyl cyanide m-chlorophenyl hydrazone (CCCP), which disrupts the mitochondrial membrane potential (ΔΨm), prevents the appearence of 30 kDa StAR in the mitochondria, which suggests that StAR import into the mitochondrial matrix and its subsequent processing are dependent on an intact ΔΨm [9], [16], [17]. After hormone stimulation, StAR is associated with a mitochondrial multiprotein complex [18]. In this regard, Rone and co-workers have recently described a 800-kDa mitochondrial bioactive complex named “transduceome”, containing the OMM translocator protein (18 kDa, TSPO) and the voltage-dependent anion channel (VDAC) along with other proteins, essential for cholesterol metabolism [19]. Particularly, VDAC1 interacts with bioactive phosphorylated StAR at the OMM, facilitating its activity [20].

It is well known that cAMP-dependent protein kinase (PKA) activation increases StAR gene transcription [21], [22], [23] and other kinases, such as ERK1/2, regulating steroidogenesis by genomic and non-genomic effects [24], [25], [26], [27], [28], [29]. Several groups, including ours, have shown that ERK1/2 and MEK1/2 are targeted to the mitochondria in different tissues, particularly at the OMM [30], [31] and that mitochondrial ERK1/2 is a cholesterol transport regulator modulating StAR phosphorylation [28].

Mitochondrial fusion/fission events are critically important for maintaining the integrity of these organelles [32]. Two dynamin-like GTPases, Mitofusin (Mfn) 1 and 2, are involved in mitochondrial fusion. They are implicated in the modulation of mitochondria-mitochondria and endoplasmic reticulum (ER)-mitochondria interactions. We have previously shown that steroid synthesis involves both types of fusion, the association between mitochondria and between mitochondria and ER membranes, known as MAM (mitochondria-associated membranes) [33]. Regulation of cholesterol transport implicates fusion of mitochondria through the increase of mitochondrial Mfn2, along with StAR phosphorylation by PKA and ERK [28], [33], [34], [35].

In this study, we evaluated the contribution of mitochondrial fusion and ERK phosphorylation in StAR synthesis and mitochondrial localization. We observed that StAR activity depends on the correct activation and localization of mitochondrial ERK in the temporal frame of kinase activity. We showed for the first time that StAR depends on mitochondrial fusion to reach the mitochondria where it is retained through a mechanism involving ERK phosphorylation in the presence of cholesterol. Here, we highlight the fact that StAR site of action is effectively the OMM, which establishes a StAR activity cycle that could in turn enable the metabolism of many molecules of cholesterol *per* unit of StAR protein.

## Materials and Methods

### Materials

Purified hCG was provided by Dr. Parlow (National Hormone and Pituitary Program, National Institute of Diabetes & Digestive & Kidney Diseases; NIDDK, NIH, Bethesda, MD, USA). Waymouth MB752/1 cell culture media, acrylamide, bis.acrylamide, agarose, BSA, 8-bromoadenosine 3′∶5′-cyclic monophosphate (8Br-cAMP), H89, 22(R)-OH-cholesterol and carbonyl cyanide m-chlorophenyl hydrazone (CCCP) were purchased from Sigma Chemical Co. (St. Louis, MO, USA). Serum, antibiotics, trypsin-EDTA and Lipofectamine 2000 were from Life Technologies, Inc. (Gaithersburg, MD, USA). Tri-Reagent was from Molecular Research Center Inc. (Cincinnati, USA). Electrophoresis supplies, polyvinylidendifluoride membrane (PVDF) and secondary antibody (horseradish peroxidase conjugated goat antibody) were from Bio-Rad Laboratories Inc. (Hercules, CA, USA). Antibodies against Mfn2 and StAR FL (full length) were obtained from Santa Cruz Biotechnology Inc. (Dallas, Texas, USA). Anti-OxPhos complex III core 2 subunit (III Complex) was purchased from Invitrogen (Carlsbad, CA, USA). M-MLV reverse transcriptase (RT), GoTaq DNA polymerase and other molecular biology reagents were purchased from Promega (Madison, WI, USA). PD98509 was obtained from Calbiochem (San Diego, CA, USA). SYBR Select Master Mix was obtained from Applied Biosystems (Carlsbad, CA, USA). Sterile and plastic material for tissue culture was from Orange Scientific (Braine-l’Alleud, Belgium). All other reagents were of the highest grade available.

### Cell Culture

The MA-10 cell line is a clonal strain of mouse Leydig tumor cells that produces progesterone (P4) rather that testosterone as the main steroid [36]. MA-10 cells were generously provided by Mario Ascoli from the University of Iowa, College of Medicine (Iowa City, IA) [36] and were handled as described previously [28]. Cells were maintained in Waymouth MB752/1 growth medium, containing 1.1 g/l NaHCO_3_, 20 mM HEPES, 50 µg/ml gentamicin and 15% heat-inactivated horse serum. Cells were maintained at 36°C in a humidified atmosphere containing 5% CO_2_. Purified hCG with a biological potency of 11900 IU/mg or 8Br-cAMP, a permeable analog of cyclic-AMP, were used to treat the cells for the indicated times.

### Cell Transfection and Plasmid Constructions

Cells were transiently transfected using Lipofectamine 2000 reagent and transfection was performed with pRc/CMVi plasmid either containing StAR wild-type cDNA (StAR wt) or StAR mutated cDNA (StAR S232A), as we used previously [28]. In another set of experiments, cells were transfected with pSUPER.retro plasmid containing the Mfn2-shRNA as described previously [33]. In all cases, empty vectors were used as control (mock transfection).

### Phosphorylation and Import/translocation Assay

1.5 µg of the pRc/CMVi plasmid containing cDNA sequence from StAR wt and StAR S232A were transcribed/translated *in* *vitro* using the Rabbit Reticulocyte Lysate System (Promega Corp. Madison, WI, USA). Plasmids were incubated separately in 70 µl of the master mix, supplemented with L-Methionine as indicated by the manufacturer, plus 300 µg of mitochondrial fraction obtained from control MA-10 cells in import buffer, as previously described [37]. After 30 min incubation at 30°C, 1 µg of constitutively active His-tagged ERK1 (Calbiochem-Millipore, MA, USA) in the phosphorylation mixture (20 mM MOPS pH 7.5, 10 mM MgCl_2_, 5 mM EGTA, 2×10^−4%^ Tween-20, 100 µM ATP, 1 mM sodium orthovanadate, 1 mM DTT) was added plus 10 µCi of [γ-^32^P]ATP and 50 µM of cholesterol. The reaction continued for other 30 min at 30°C and was finished after 1 h by the addition of 25 µM FCCP for 10 min. at 4°C. The samples were centrifuged at 20000*×g* for 20 min and the mitochondrial pellet was resuspended in MSHE buffer, as previously described [28]. Then, mitochondrial fraction was separated by SDS-PAGE electrophoresis. The PVDF membranes were subjected to autoradiography and immunoblots were performed using antibodies anti-pERK and anti-StAR.

### Cell-free Assay for Mitochondrial Steroidogenesis

Cells were treated as indicated in the corresponding figure. Then, subcellular fractionation and measurement of mitochondrial steroidogenesis was performed according to our published procedures [28]. Mitochondria were incubated in the presence of cholesterol (50 µM) as substrate for 30 min at 30°C. Incubations were performed in the absence or presence of 1 µg of constitutively active His-tagged ERK1 together with 1 IU of PKA catalytic subunit (Sigma Chemical Co., St. Louis, MO, USA). Then, P4 production in the incubation media was determined by radioimmunoassay [33].

### Protein Quantification and Western Blot

Mitochondria were isolated as described previously [38]. Mitochondrial proteins were determined by Bradfords method [39] and were subjected to SDS-PAGE as described previously [40]. Membranes were then incubated with the primary antibodies anti-Mfn2 (1∶1000) [33], anti-phospho ERK (1∶1000) or anti-StAR (1∶1000) [28]. Then, anti-III Complex (1∶10000) and anti-total ERK antibodies (1∶1000) were used to normalize the results [33].

### RNA Extraction, Semi-quantitative RT-PCR and Real-time PCR

MA-10 Leydig cells total RNA was extracted using Tri-Reagent following the manufacturer’s instructions. Two µg of total RNA were reverse transcribed using random hexamers and M-MLV Reverse Transcriptase according to the manufacturer's protocol.

For semi-quantitative RT-PCR, the following specific primers were used: mouse StAR cDNA forward, 5′-GGGACGAAGTGCTAAGTAAGATGG-3′ and reverse, 5′-GGTCAATGTGGACAGTCC-3′ (amplicon size 566-bp); and mouse ribosomal protein L19 cDNA forward, 5′-GAAATCGCCAATGCCAACTC-3′ and reverse, 5′-TCTTAGACCTGCGAGCCTCA-3′ (amplicon size 405-bp) [41]. Primers were obtained from Invitrogen (Carlsbad, CA, USA). Reaction conditions were one cycle at 94°C for 5 min, followed by 22 cycles (for StAR) or 23 cycles (for L19) at 94°C for 30 sec, 55°C for 30 sec, and 72°C for 30 sec, and one final cycle at 72°C for 10 min. The number of cycles used was optimized for each gene to fall within the linear range of PCR amplification. PCR products were resolved on a 1.5% (wt/vol) agarose gel containing 0.5 µg/ml of ethidium bromide to determine the molecular sizes of the StAR and L19 amplicons. Gel images were acquired with a GelPro analyzer (IPS, North Reading, MA). Levels of StAR and L19 mRNA were quantified with a computer-assisted image analyzer (ImageQuant 5.2) and PCR results for each sample were normalized by L19 mRNA as an internal control.

For Real-Time PCR, the following specific primers were used: mouse StAR cDNA forward, 5′-TTGGGCATACTCAACAACCA-3′ and reverse, 5′-CTTGACATTTGGGTTCCAC-3′ [42]; and mouse 18S RNA forward, 5′-ATTCCGATAACGAACGAGACT-3′ and reverse, 5′-AGCTTATGACCCGCACTTACT-3′ (obtained from RealTimePrimers.com, Elkins Park, PA, USA). Real-time PCR was performed using Applied Biosystems 7300 Real-Time PCR System. For each reaction, 20 µl of solution containing 5 µl of cDNA, 10 µM forward and reverse primers, and 10 µl of SYBR Select Master Mix was used. All reactions were performed in triplicate. Amplification was initiated by a 2-min preincubation at 50°C, 2-min incubation at 95°C, followed by 40 cycles at 95°C for 15 sec, 55°C for 15 sec and 72°C for 1 min, terminating at 95°C for the last 15 sec. StAR mRNA expression levels were normalized to mouse 18S RNA expression, performed in parallel as endogenous control. Real-time PCR data were analyzed by calculating the 2^−ΔΔCt^ value (comparative Ct method) for each experimental sample.

### Radioimmunoanalysis [33] and Statistics

P4 production in cell culture media was measured by RIA as described previously [40]. Statistical significance was determined by Student’s *t* test or analysis of variance (ANOVA) followed by Student-Newman-Kuels test.

## Results

### Maximal Steroid Synthesis Depends on Mitochondrial Mediators Supporting StAR Function

**Figure 1:**
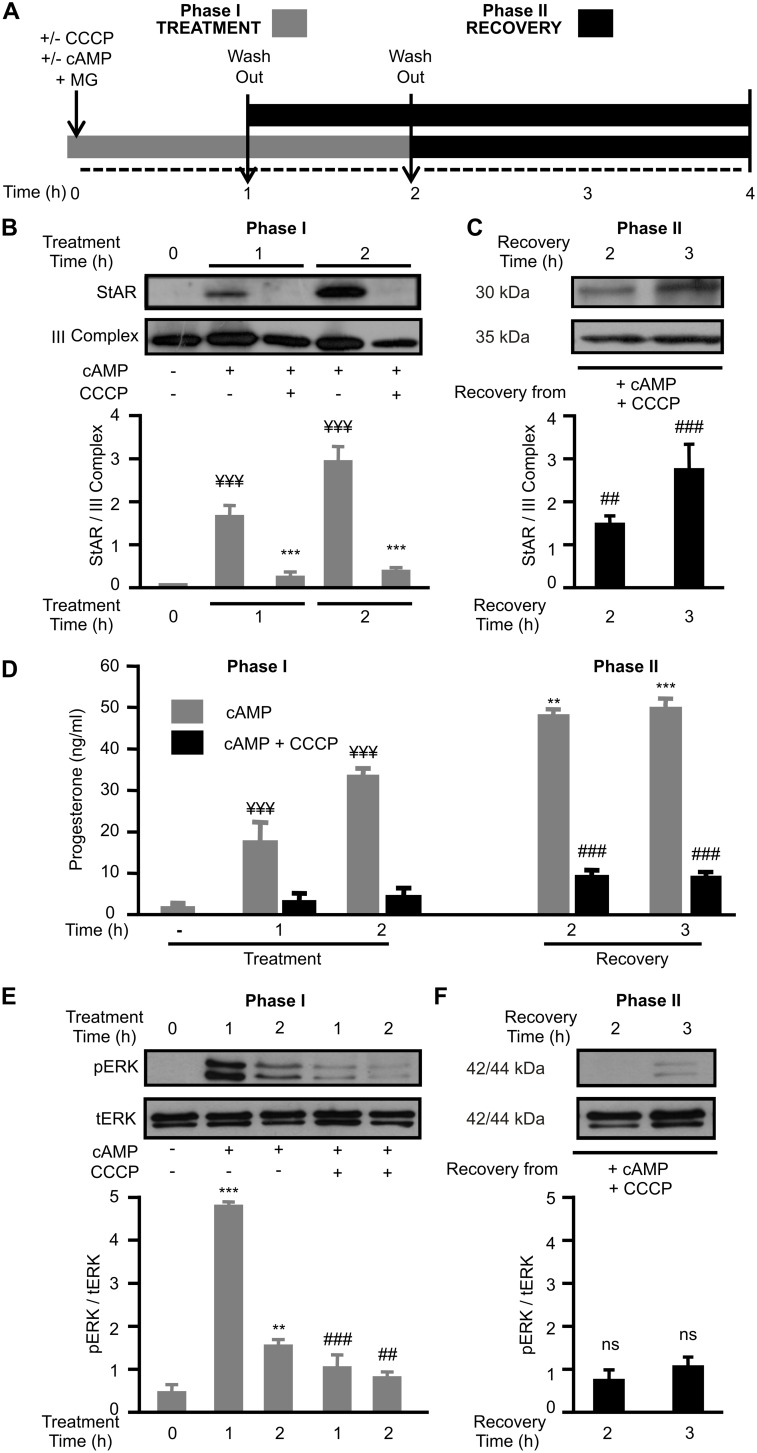
Mitochondrial StAR protein *per se* is not enough to sustain steroidogenesis stimulated by cAMP. Design of two-phase experiments. **A.** Cells were incubated in the absence or presence of 8Br-cAMP (cAMP) (1 mM) and CCCP (5 µM) for 1 h or 2 h (Phase I; Treatment). Here, the proteasome inhibitor MG132 (MG; 5 µM) was used to avoid cytoplasmatic StAR degradation. Then, these additives were removed (wash-out) with fresh media (Phase II; Recovery). Among cells incubated with cAMP plus CCCP, those treated for 2 h in Phase I recovered after 2 h in Phase II, while those treated for 1 h in Phase I recovered after 3 h in Phase II, reaching in both cases a 4 h final experimental time. **B.** Mitochondrial proteins from cells in Phase I were obtained and western blotting was performed. Membranes were sequentially blotted for StAR and III Complex. A representative western blot is shown. For each band, the OD of expression levels of StAR protein were quantified (arbitrary units) and normalized to the corresponding III Complex protein. The relative levels of StAR protein are shown: ¥¥¥ *p*<0.001 vs. control; ***p<0.001 vs. cAMP alone. **C.** In another set of experiments, cells were sequentially subjected to Phase I and II, thereafter mitochondrial proteins were isolated and western blotting was performed as indicated in panel B. The relative levels of StAR protein are shown: ### *p*<0.001 vs. 1 h cAMP +CCCP in Phase I; ## *p*<0.01 vs. 2 h cAMP +CCCP in Phase I. **D.** P4 production in the culture media from Phase I and II was measured by RIA and data are shown as P4 concentration (ng/ml): ¥¥¥ *p*<0.001 vs. control; ***p<0.001 vs. cAMP alone in Phase I, **p<0.01 vs. cAMP alone in Phase I, ### *p*<0.001 vs. recovery from cAMP alone. **E.** Mitochondrial proteins from cells in Phase I were obtained and western blotting was performed. Membranes were sequentially blotted for phospho-ERK1/2 (pERK) and total ERK1/2 (tERK). A representative western blot is shown. For each band, the OD of expression levels of pERK were quantified (arbitrary units) and normalized to the corresponding tERK protein. The relative levels of pERK are shown: ***p<0.001 vs. control, **p<0.01 vs. control, ### *p*<0.001 vs. 1 h cAMP alone, ## *p*<0.01 vs. 2 h cAMP alone. **F.** In another set of experiments, cells were sequentially subjected to Phase I and II, thereafter mitochondrial proteins were isolated and western blotting was performed as indicated in panel **E.** The relative levels of pERK are shown: ns *p*>0.05 vs. cAMP +CCCP in Phase I. Results are expressed as mean ± SEM of three independent experiments.

CCCP mediated-ΔΨm disruption effects on StAR metabolism, mitochondrial import and steroidogenesis after hormone stimulation have been previously shown in MA-10 Leydig cells [9], [16], [43]. The well-known effects of this protonophore on membrane potential, mitochondrial import qualities and normal mitochondrial dynamics are entirely reversible [33], [44], [45]. Even though the effect of CCCP removal on StAR processing and localization has already been described [16], [17], there is no evidence of the recovery of steroid synthesis after the re-establishment of the ΔΨm. Therefore, we evaluated mitochondrial StAR and steroidogenesis after inhibition and subsequent restoration of the ΔΨm. To address this issue, we performed a two-phase experiment, as shown in Figure 1A. In Phase I (Treatment), MA-10 cells were stimulated with 8Br-cAMP (cAMP) in the presence or absence of CCCP. In Phase II (Recovery), after CCCP wash-out, fresh medium was added to remove both the stimulant and the uncoupling agent. Phase II measures the capacity of newly synthesized StAR (generated in Phase I) to localize efficiently in mitochondria after ΔΨm is restored. Among cells incubated with cAMP plus CCCP, those treated for 2 h in Phase I recovered after 2 h in Phase II, while those treated for 1 h in Phase I recovered after 3 h in Phase II, reaching in both cases a 4 h final experimental time (Figure 1A). As seen in Figure 1B, the levels of mitochondrial StAR are clearly detected by immunoblot after 1 and 2 h stimulation with cAMP (see second and forth lanes), but very low StAR levels are observed after 1 and 2 h stimulation with cAMP in the presence of CCCP (see third and fifth lanes). Figure 1C shows mitochondrial StAR recovery after CCCP removal in Phase II, with a clear presence after 2 h and robust increase after 3 h (Fig. 1C, Recovery Time 2 and 3 h). The inhibitory effect of CCCP and its recovery in Phase II are also observed in StAR mRNA levels (Figure S1).

Next, we analyzed the impact of the two-phase assay on cholesterol metabolism, measured as P4 production. Figure 1D shows that, as expected, P4 production in the presence of cAMP is abrogated by CCCP at every time analyzed in Phase I (left half panel) and not reactivated after CCCP elimination (right half panel) in Phase II, even when immunoblot revealed StAR presence in the mitochondria. In turn, the stimulation of steroid synthesis sustained by 22(R)-OH-cholesterol (a permeable analog of cholesterol) was not statistically different in the presence or absence of CCCP. These findings, shown in Table S1, indicate that P4 synthesis inhibition in the presence of CCCP is not due to mitochondrial enzyme damage.

Given that mitochondrial StAR is not enough to reactivate complete steroidogenesis, these results suggest that steroid production mediated by StAR involves the cooperation with (an)other factor(s) in the mitochondria of steroidogenic cells, particularly in Phase I. To adress this particular issue, we analyzed phospho-ERK1/2 (pERK) levels in mitochondria after the two-phase experiment. Figure 1E shows that, in agreement with previous work (Duarte et al., 2012), mitochondrial cAMP-stimulated pERK diminished in the presence of CCCP (see forth and fifth lanes). Interestingly, Figure 1F shows that mitochondrial pERK levels were not restored either after 2 or 3 h of Phase II (Recovery Time 2 and 3 h). These results are completely in line with the lack of steroid synthesis in Phase II, given that mitochondrial ERK activity is required for steroidogenesis [26], [28].

**Figure 2:**
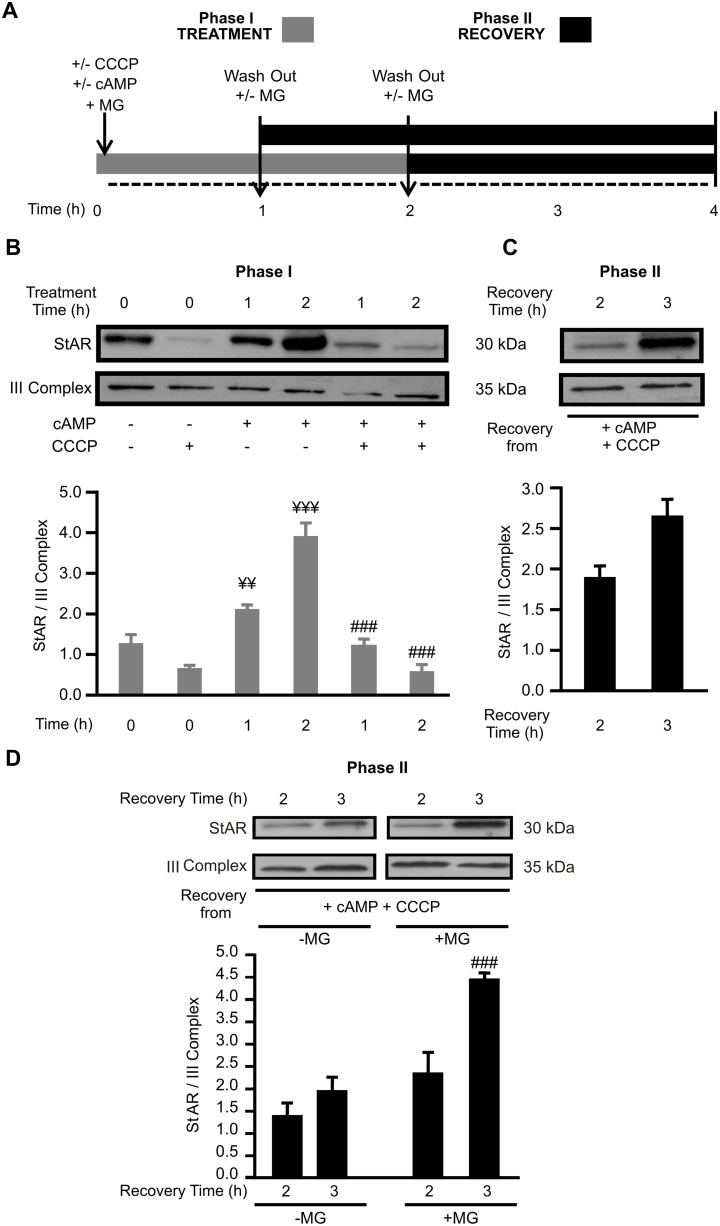
StAR mitochondrial levels depend on post-transcriptional events. Cells were transfected with a pRc/CMVi vector containing the cDNA of wild type StAR full length (StAR wt). **A.** Cells were incubated in the absence or presence of 8Br-cAMP (cAMP) (1 mM) and CCCP (5 µM) for 1 h or 2 h (Phase I; Treatment). Here, the proteasome inhibitor MG132 (MG; 5 µM) was used to avoid cytoplasmatic StAR degradation. Then, these additives were removed (wash-out) with fresh media (Phase II; Recovery). Among cells incubated with cAMP plus CCCP, those treated for 2 h in Phase I recovered after 2 h in Phase II, while those treated for 1 h in Phase I recovered after 3 h in Phase II, reaching in both cases a 4 h final experimental time. **B.** Mitochondrial proteins from cells in Phase I were obtained and western blotting was performed. Membranes were sequentially blotted for StAR and III Complex. A representative western blot is shown. For each band, the OD of expression levels of StAR protein were quantified (arbitrary units) and normalized to the corresponding III Complex protein. The relative levels of StAR protein are shown: ¥¥¥ *p*<0.001 and ¥¥ *p*<0.01 vs. control; ### *p*<0.001 vs. cAMP alone. **C.** In another set of experiments, cells were sequentially subjected to Phase I and II, thereafter mitochondrial proteins were isolated and western blotting was performed as indicated in panel B. The relative levels of StAR protein are shown. **D.** cAMP stimulated- and CCCP treated-cells were incubated with or without MG132 (5 µM) during Phase II. Mitochondrial proteins were obtained and western blotting was performed as indicated in panel B: ### *p*<0.001 vs. 3 h Recovery Time (Phase II) without MG. Results are expressed as mean ± SEM of three independent experiments.

In order to analyze the consequence of ΔΨm disruption in mitochondrial StAR protein levels, we performed a transient transfection over-expressing StAR (Figure 2), so that the observed effect of CCCP on endogenous StAR mRNA levels can be excluded (Figure S1) and post-transcriptional effects can be evaluated. The experimental protocol (Fig. 2A) was the same as for Figure 1A. Figure 2B shows that the exogenous StAR is localized in the mitochondria, even in absence of stimulation (see first lane). The addition of CCCP abrogates the association of StAR to mitochondria both in absence (see second lane) and presence of cAMP stimulation (see fith and sixth lanes). Figure 2C shows that mitochondrial StAR is recovered after CCCP wash-out in Phase II in a time-dependent manner (Fig. 2C, Recovery Time 2 and 3 h). Taken together, the results presented so far reinforce the fact that StAR is not completely effective by itself for reactivating P4 synthesis and that mediators, probably active ERK, are needed in Phase I to elicit maximal steroid synthesis after mitochondrial StAR levels are restored.

Interestingly, the presence of MG132 (a proteasome inhibitor) in Phase II increases StAR mitochondrial levels two-fold (Fig. 2D, Recovery Time 2 and 3 h, right panel with MG), as compared to levels observed in absence of MG132 (left panel, without MG). Results were compared within Figure 2D, with the same gel and autoradiography exposure time. This result suggests that the absence of mitochondrial mediator/s due to the presence of CCCP in Phase I makes StAR more susceptible to mitochondrial proteases, as one of the mechanisms involved in the regulation of mitochondrial StAR levels.

### ERK-mediated Phosphorylation of StAR is Required to Retain the Protein in Mitochondria

**Figure 3:**
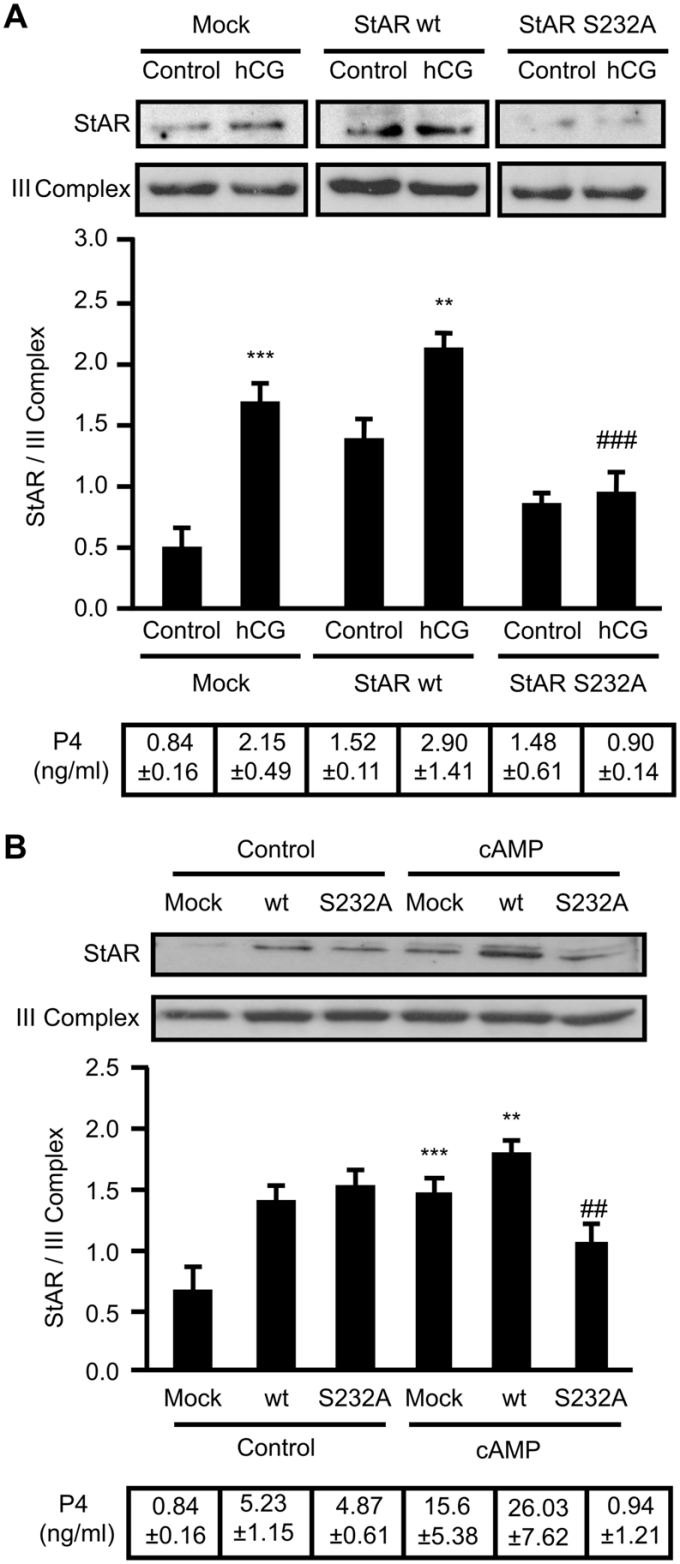
ERK phosphorylation of StAR is required for the correct association of StAR with the mitochondria. Cells were transfected with an empty pRc/CMVi vector (mock) or containing the StAR wt cDNA (StAR wt) or StAR mutant form (StAR S232A). After 48 h, cells were stimulated for 1 h with **A.** hCG (20 ng/ml) or **B.** 8Br-cAMP (cAMP) (0.5 mM). Membranes were sequentially blotted for StAR and III Complex. Representative western blots are shown. For each band, the OD of the expression levels of StAR protein were quantified (arbitrary units) and normalized to the corresponding III Complex protein. The relative levels of StAR protein are shown: **A.** ***p<0.001 vs. control mock; **p<0.01 vs. control StAR wt; ### *p*<0.001 vs. hCG StAR wt. **B.** ***p<0.001 vs. control mock; **p<0.01 vs. control StAR wt; ## *p*<0.01 vs. cAMP StAR wt. Results are expressed as mean ± SEM of three independent experiments. Progesterone production was determined by RIA in the incubation media. Results are indicated in a Table at the bottom of each panel. Data are expressed as ng/ml of P4.

StAR is phosphorylated by ERK *in* *vitro* in serine 232 (Ser232), a crucial event in StAR activity and cholesterol transport. Mitochondrial ERK phosphorylation was followed by progressive decrease of its activity during the first hour of hormonal action [28]. As seen in this work, ΔΨm is required for mitochondrial StAR localization; nevertheless, P4 production achieved after the recovery of ΔΨm and StAR translocation is not consistent with a fully activated protein. The inhibition of ΔΨm with CCCP partially blocks the association of ERK with the mitochondria after hormone stimulation [33] and also inhibits cAMP-provoked phosphorylation of total ERK [46] in MA-10 Leydig cells. As ERK phosphorylation is not recovered after CCCP wash-out, we examined the role of ERK phosphorylation in StAR localization/retention in mitochondria and steroid synthesis. For this purpose, we transiently transfected cells with StAR wt and StAR S232A, a mutant form of StAR in which the Ser232 was converted to Alanine, a non-phosphorylatable amino acid. Over-expression of the StAR wt correlates with the major mitochondrial StAR content, as observed by immunoblot even in basal cellular conditions (Figure 3A and B). After hCG-stimulation of cells for 1 h, mitochondrial StAR wt levels increase in the mitochondria, but not those of the StAR S232A form, indicating that StAR S232A mutation partially blocks the accessibility of StAR to the mitochondria (Fig. 3A). The degree of inhibition exerted by the StAR S232A form correlates with the transfection efficiency rates (Poderoso et al., 2008) and, as similar results were obtained with 1 h-cAMP stimulation (Figure 3B), this effect might be mediated by upstream PKA activity. These results suggest that StAR phosphorylation by ERK is needed for mobilization or retention of this protein on the OMM. As previously described [28], over-expression of StAR S232A significantly inhibits P4 production after hormone stimulation (bottom of each panel).

**Figure 4:**
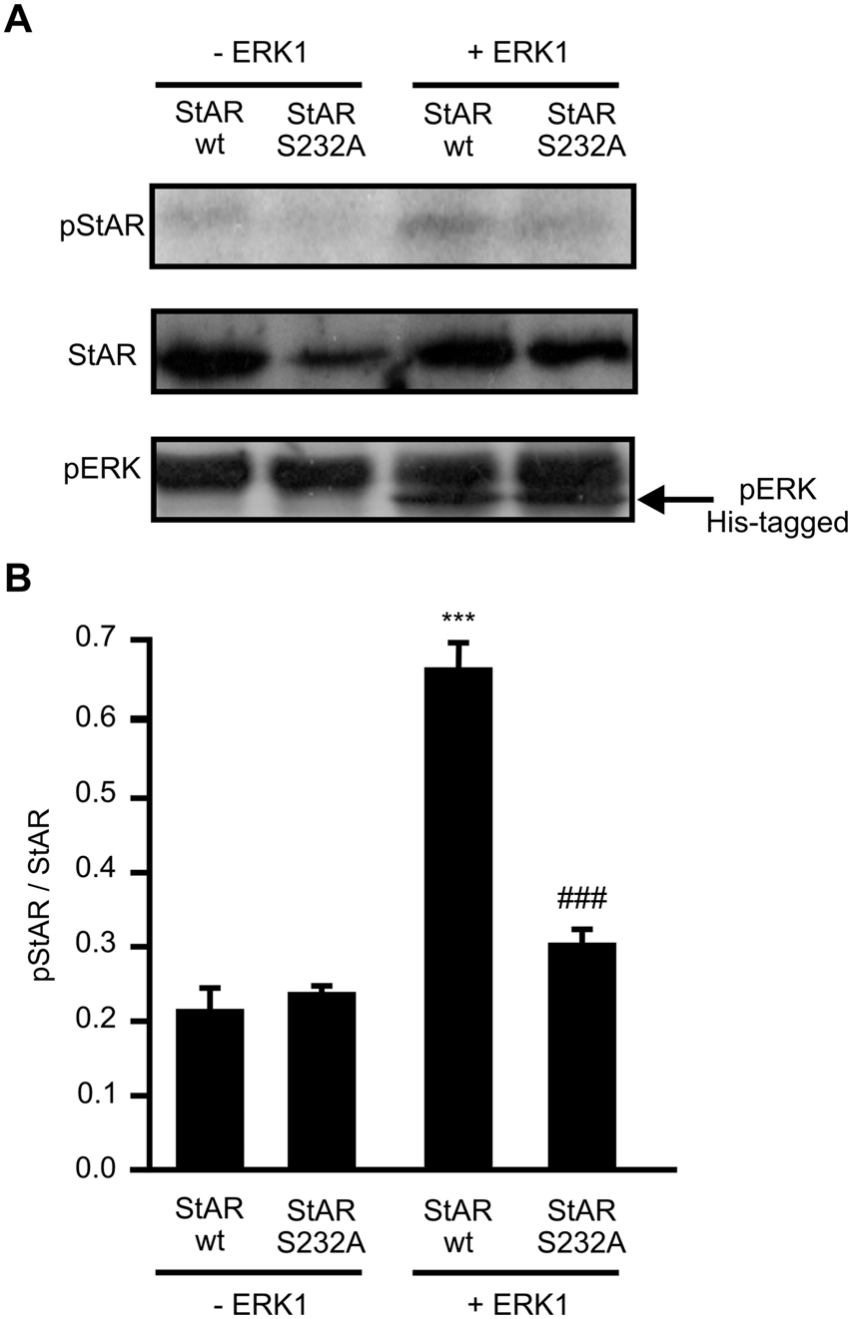
ERK phosphorylation of StAR in Ser232 drives StAR retention on the OMM. pRc/CMVi vector containing the StAR wt cDNA (StAR wt) or StAR mutant form (StAR S232A) were subjected to a transcription/translation import assay and *in vitro* phosphorylation (see Materials and Methods). Mitochondrial proteins were separated by SDS-PAGE, transfered to a PVDF membrane and then autoradiography and immunoblot were performed. **A.** Levels of phosphorylated StAR (pStAR) are shown in the presence or absence of active ERK1 (upper panel). The autoradiography displays a representative result of three independent experiments. The immunoblots (lower panels) show total mitochondrial StAR and phospho-ERK (pERK) levels. Representative western blots are shown. **B.** For each band, the OD of pStAR levels were quantified (arbitrary units) and normalized to the corresponding total StAR protein levels. The relative levels of pStAR are shown: ***p<0.001 vs. StAR wt without ERK1; ### *p*<0.001 vs. StAR wt with ERK1. Results are expressed as mean ± SEM of three independent experiments.

To confirm that ERK phosphorylation of StAR is effectively the key event involved in StAR retention at the OMM, we performed a mitochondrial import and phosphorylation *in* *vitro* assay. Plasmids containing StAR wt and StAR S232A sequences were subjected to the import assay in the presence of isolated mitochondria from control cells and recombinant active ERK1. Then, mitochondrial proteins were subjected to SDS-PAGE, autoradiography and immunoblot. In Figure 4 we showed that there is a slight mitochondrial signal of phospho-StAR wt in the absence the kinase, probably due to some extent of endogenous mitochondrial ERK. Practically, no signal of phosphorylation of StAR S232A is observed. The addition of recombinant active ERK1 provokes a three fold increase in phospho-StAR wt signal meanwhile StAR S232A signal is not affected. An immunoblot against pERK is shown to asses the presence of active ERK1 in the mitochondrial fraction and mitochondrial endogenous ERK, as mentioned above.

### Active ERK1/2 and PKA are Required Along with Mitochondrial StAR in Order to Attain a Maximal Steroidogenesis Rate in Mitochondria

**Figure 5:**
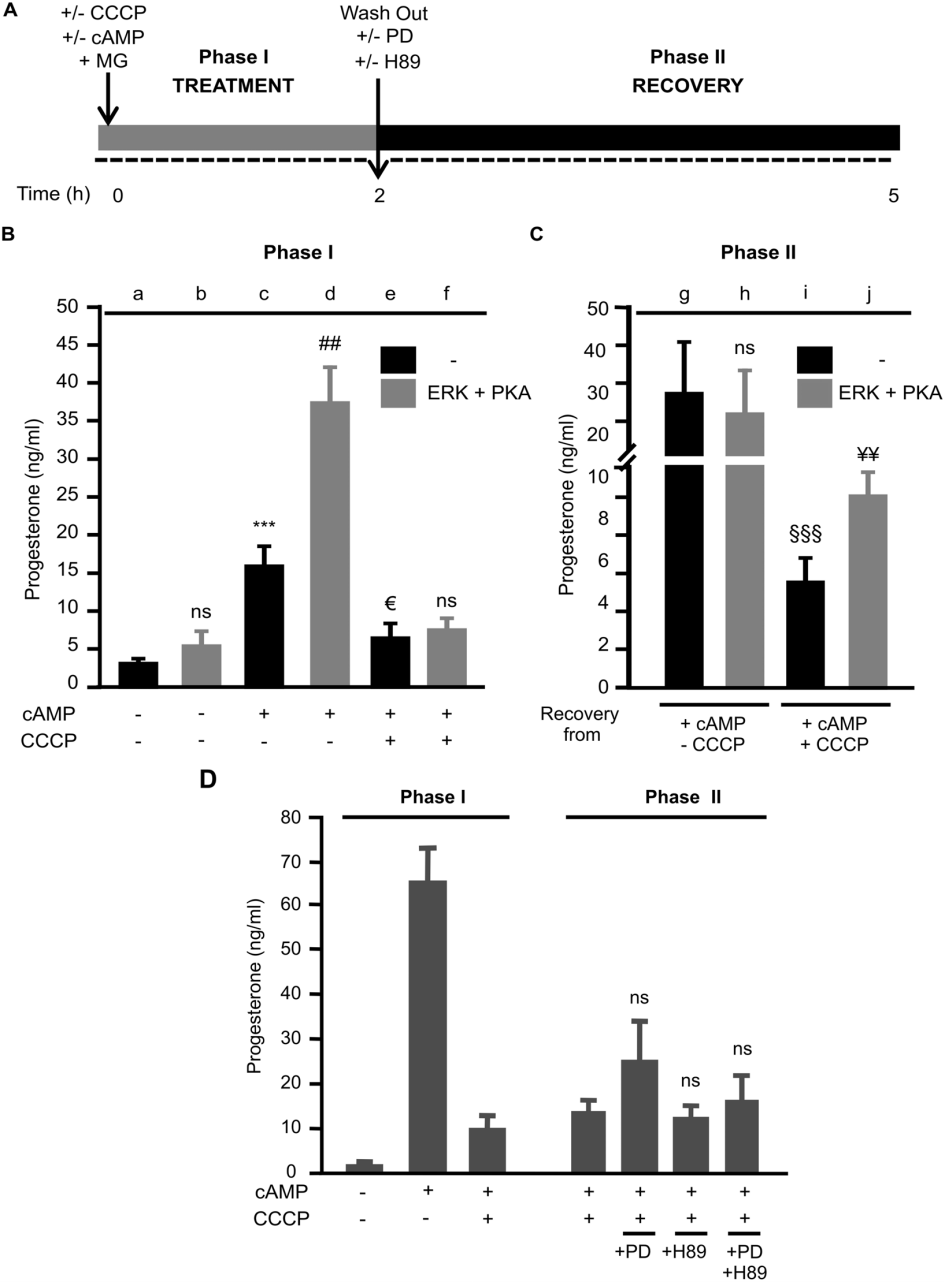
ERK and PKA are strictly required as mediators to achieve maximal steroidogenesis in the presence of mitochondrial StAR. **A.** Design of two-phase experiments. Cells were incubated in the absence or presence of 8Br-cAMP (cAMP) (1 mM) and CCCP (5 µM) for 2 h (Phase I; Treatment). Here, the proteasome inhibitor MG132 (MG; 5 µM) was used to avoid cytoplasmatic StAR degradation. Then, these additives were removed (wash-out) with fresh media (Phase II; Recovery) for 3 h. For panels B and C, mitochondria from cells subjected to the two-phase assay were isolated and incubated, with cholesterol (50 µM) as substrate, in the presence (grey bars) or absence (black bars) of constitutively active His-tagged ERK1 together with PKA catalytic subunit. After the above incubations, mitochondria were pelleted, media was collected and mitochondrial P4 production was measured by RIA. Data are shown as P4 concentration (ng/ml): **B.** Phase I: ns *p*>0.05 b vs. a; ***p<0.001 c vs. a; ## *p*<0.01 d vs. c; € *p*<0.05 e vs. c; ns *p*>0.05 f vs. e. **C.** Phase II: ns *p*>0.05 h vs. g; §§§ *p*<0.001 i vs. g; ¥¥ *p*<0.01 j vs. i. Results are expressed as mean ± SEM of three independent experiments. **D.** In another set of experiments, cells were sequentially subjected to Phase I and II. During Phase II, incubation was conducted in the presence or absence of H89 (20 µM) or PD98059 (PD; 50 µM). Then, media was collected and P4 production was measured by RIA. Data are shown as P4 concentration (ng/ml): ns *p*>0.05 with inhibitors alone or both vs. without inhibitors.

Steroidogenesis requires full activity of ERK and PKA localized at the OMM’s transduceome complex [19], [30], [31]. Also, mitochondrial ERK is transiently activated after hormone stimulation [28]. Hence, and once we had proven that StAR requires ERK activity to localize correctly in the mitochondrial context, the question remained whether both ERK and PKA could contribute to StAR’s full activity through its retention on the OMM. To address this issue, we performed a cell-free assay, whose experimental protocol is indicated in Figure 5A. In Phase I (Fig. 5B) we observed basal P4 production by mitochondria from unstimulated cells with a small increase after addition of recombinant active ERK1 and PKA catalytic subunit (bar a vs. b), which is probably due to a small amount of mitochondrial StAR. P4 ratio was higher in mitochondria from 2 h-cAMP-stimulated cells (bar c vs. a); moreover, steroid synthesis from these isolated mitochondria was significantly and robustly augmented in the presence of recombinant active ERK1 and PKA catalytic subunit (bar d vs. c). These results indicate that, even when StAR is present after 2 h of cAMP stimulation, ERK and PKA activity are strongly required to achieve maximal steroid production in this period of time. In contrast, P4 production was abrogated when mitochondria were isolated from stimulated cells in the presence of CCCP (bar e vs. c); since CCCP prevents StAR from reaching the OMM, neither ERK nor PKA had effects on P4 synthesis (bar f vs. e). Regarding Phase II (Fig. 5C), when cells were stimulated for 2 h in the presence of cAMP alone (see bar c), mitochondrial P4 synthesis was maximal in the culture media collected after 3 h (see bar g), as a consequence of remnant cAMP stimulation in the first 2 h. The addition of ERK and PKA fail to elicit significant difference in P4, which was probably to the maximal effect provoked by cAMP in the transduction signal system (bar h vs. g). We observed that, although CCCP was eliminated for 3 h in Phase II, mitochondrial P4 synthesis was not reactivated (bar i vs. g). This result mimics those shown in Figure 1D obtained in whole cells. However, the incubation of isolated mitochondria (from cells treated with cAMP and CCCP) with the two constitutively active kinases yielded a moderate but significant increase in P4 production (bar j vs. i). The strict requirement of active ERK and PKA was confirmed using specific inhibitors of both pathways, PD98059 (PD) and H89 respectively. Since these compounds are not expected to affect constitutively active kinases, we used them in whole cells. Figure 5D shows that the addition of H89 or PD98509 to cells incubation media in Phase II (see right panel) did not elicit any significant change in the low rate of P4 synthesis achieved after CCCP wash-out. The effect of ERK inhibition is not significant compared to the recovery without PD98509 since active ERK is not present in mitochondria in this condition. It might be suggested that PKA activity exhibits a similar pattern, dependending on the observed effect with H89, or with the combination of both compounds. Left panel of the Fig. 5D displays Phase I, as already described in Figure 1.

### Mitochondrial Fusion is Associated with the Efficient Localization of StAR and ERK Activity in Mitochondria

**Figure 6:**
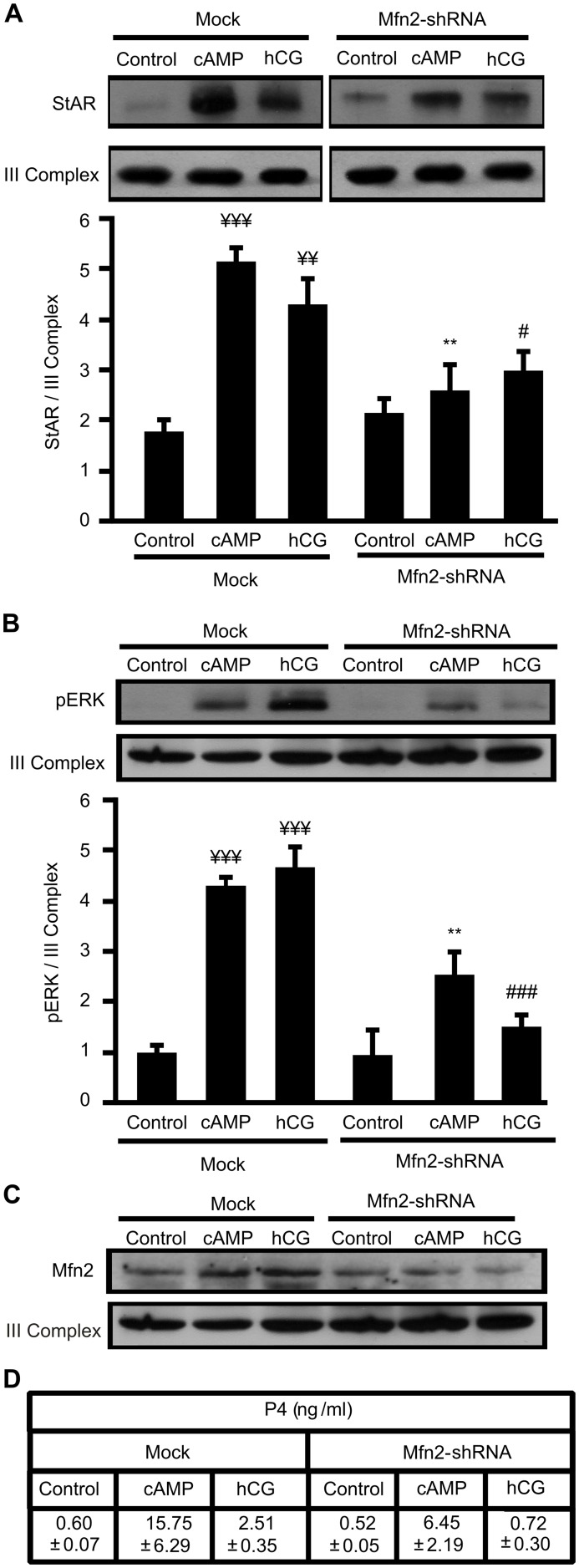
Mfn2 protein is necessary for StAR and pERK mitochondrial localization. Cells were transfected with an empty pSUPER.Retro vector (mock) or containing a Mfn2-shRNA. After 48 h, cells were stimulated with hCG (20 ng/ml) or 8Br-cAMP (cAMP) (0.5 mM) for 1 h. Mitochondrial proteins were obtained and western blotting was performed. Membranes were sequentially blotted with anti-StAR, phospho-ERK (pERK) or anti-Mfn2 antibodies, and III Complex antibody as loading control. An image of a representative western blot is shown. For each band, the OD of expression levels of StAR and pERK proteins were quantified (arbitrary units) and normalized to the corresponding III Complex protein. **A.** The relative levels of StAR protein are shown: ¥¥¥ *p*<0.001 and ¥¥ *p*<0.01 vs. control mock; **p<0.01 vs. cAMP mock; # *p*<0.05 vs. hCG mock. **B.** The relative levels of pERK protein are shown: ¥¥¥ *p*<0.001 vs. control mock; **p<0.01 vs. cAMP mock; ### *p*<0.001 vs. hCG mock. Results are expressed as mean ± SEM of three independent experiments.**C.** An image of a representative western blot is shown to assess shRNA knockdown efficiency. **D.** Progesterone production was determined by RIA in the incubation media. Data are expressed as ng/ml of P4.

We recently described that Mfn2 is up-regulated by the hCG/cAMP system in MA-10 Leydig cells and postulated a key role of mitochondrial fusion in the hormone stimulation of steroidogenesis [33]. Although StAR is a mitochondrial protein and mitochondrial fusion is essential for steroid synthesis, data referring to the involvement of this fusion and the dynamic patterns of StAR are not available. To determine whether StAR mitochondrial localization could, at least in part, be mediated by mitochondrial fusion, we knocked down Mfn2 by the shRNA technique as described previously [33]. Since we have already validated specific Mfn2 inhibition in MA-10 cells by means of two different shRNA constructs [33], we chose one shRNA construct to perform these experiments. Immunoblot showed that the increase in StAR mitochondrial signal after hCG and cAMP 1 h-stimulation was significantly diminished in the presence of Mfn2-shRNA (Figure 6A) with no effect on StAR basal levels. Interestingly, Figure 6B shows that mitochondrial ERK activation stimulated by cAMP and hCG, detected as phosphorylation of the kinase, was significanlty diminished when Mfn2 levels were knocked down. Figure 6C shows that Mfn2 was hormonally induced, while the efficacy of the shRNA treatment in the expression of Mfn2 was demonstrated by the reduction detected in the mitochondrial content of this protein. Under these conditions P4 biosynthesis was also inhibited, as expected (Fig. 6D). These results suggest that mitochondrial fusion could be involved in: a) StAR protein mitochondrial localization, b) mRNA StAR abundance which determines total StAR protein levels or c) a combination of both effects.

### StAR mRNA Levels are Dependent on Mfn2 Presence and Mitochondrial Fusion

**Figure 7:**
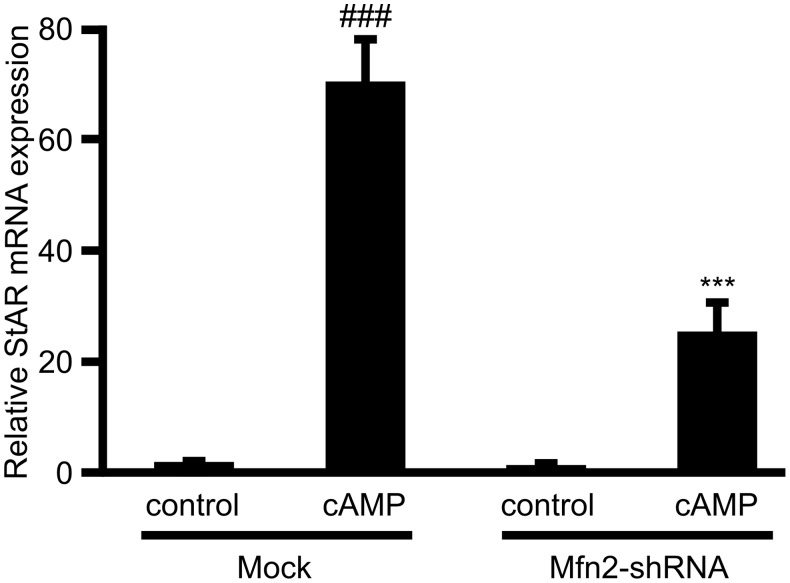
Mitochondrial fusion modulates StAR mRNA levels. Cells were transfected with an empty pSUPER.Retro vector (mock) or containing a Mfn2-shRNA. After 48 h, cells were incubated with or without 8Br-cAMP (cAMP) (0.5 mM) for 1 h. Total RNA was isolated; reverse transcribed and subjected to Real-Time PCR.using specific primers. StAR mRNA expression levels were normalized to mouse 18S RNA expression, performed in parallel as endogenous control. Real-time PCR data were analyzed by calculating the 2^−ΔΔCt^ value (comparative Ct method) for each experimental sample. The relative expression levels of StAR are shown: ### *p*<0.001 vs. control mock; ***p<0.001 vs. cAMP mock. Results are expressed as mean ± SEM of three independent experiments.

StAR gene expression regulation has been extensively studied in steroidogenic and even non-steroidogenic tissues [21], [22], [23], [47], [48]. However, little is known about post-transcriptional regulation of StAR synthesis. Previous reports indicate that stabilization of StAR mRNA is one mechanism involved in the regulation of StAR mRNA total levels [41], [49], [50]. Moreover, previous reports show that Mfn2 protein levels regulate the mRNA abundance of different proteins [51], [52]. In order to further explore the potential role of mitochondrial fusion in total cellular levels of StAR mRNA, we examined its content under hormone stimulation in Mfn2 knocked down cells. We observed that the abrogation of Mfn2 levels significantly inhibited StAR mRNA abundance after 1 h of cAMP stimulation (Figure 7). This work shows, for the first time, that mitochondrial fusion is involved, at least in part, in modulation of StAR mRNA levels in the cell.

## Discussion

In this study we have demonstrated that steroidogenesis in MA-10 Leydig cells depends on the hormonally-stimulated mitochondrial fusion that regulates StAR mRNA and protein abundance and therefore its translocation to the mitochondria. We have shown for the first time that mitochondrial ERK1/2 activation is an essential step in localization, activity and/or retention of StAR on the OMM.

We determined that StAR protein is effectively translocated to the matrix after the ΔΨm is restored, as determined by the detection of the 30 kDa processed StAR. Even though we observed a significant amount of mitochondrial StAR levels after a 2 and 3 h wash-out of CCCP, P4 production in Phase II was not reactivated in any recovery time evaluated. (Fig. 1).

P4 synthesis rate was not reactivated even when mitochondrial function was normalized and StAR localized at the matrix; then, it appears that StAR presence in the mitochondrial matrix *per se* is not sufficient to recover steroidogenesis. It is conceivable that other mitochondrial factors, which are unable to reach the mitochondria when CCCP is added to the culture media, are involved in StAR activation in Phase I. Mitochondrial ERK can be suggested as a good candidate to cooperate with StAR in Phase I to achieve maximal P4 production, since its activation/deactivation cycle correlates with the time-course of Phase I/II respectively. This notion is reinforced by the fact that mitochondrial ERK activation is impared in the presence of CCCP; nevertheless, pERK levels are not restored after CCCP wash-out in Phase II.

Since CCCP affected StAR mRNA total levels (Fig. S1) and over-expressed exogenous StAR mitochondrial presence is blocked by CCCP, results from Figures 1 and 2 together suggest that inhibition of ΔΨm affects StAR mitochondrial localization at both transcriptional and post-transcriptional level; for example, retention at the OMM.

We showed that over-expression of StAR S232A significantly abolishes the presence of mitochondrial StAR after hCG or cAMP stimulation. ERK phosphorylation affects post-transcriptionally mitochondrial StAR levels,. since expression of transfected StAR is independent of endogenous regulation. In MA-10 cells, a mitochondrial module includes MEK (Mitogen-activated protein kinase kinase), ERK and cholesterol with a direct physical association between StAR and ERK [28]. Their interaction favors StAR phosphorylation by ERK and hence could promote phospho-StAR retention in the mitochondria, particularly on the OMM where ERK resides [30]. It is the OMM localization of StAR, and not its cleavage from the 37 kDa to the 30 kDa form, that determines its activity [12]. Then, the longest StAR retention time at the OMM might render the maximal StAR activity in cholesterol transport, in agreement with previous data [14]. ERK is transiently activated after hormone stimulation in MA-10 cells and is dephosphorylated as a normal regulation pathway, mainly by the Mitogen-activated protein kinase phosphatases (MKPs) [28], [53], [54]. Interaction between phospho-StAR and ERK in mitochondria could protect ERK from dephosphorylation and inactivation. The temporal frame of ERK activity (1–2 h) in mitochondria correlates with the period of StAR major activity and cholesterol transport after hormone stimulation. These results are entirely in line with the MKP-1 down-regulation and the concomitant P4 increase after 2 h of stimulation, probably due to a long-lasting effect of active ERK in mitochondria [42]. If CCCP is present in 15 min-ACTH stimulation in Y1 adrenal cells, most of the steroid production is recovered after 15 min wash-out [9], which is consistent with full mitochondrial ERK activity.

Results depicted in Figures 4 and 5 clearly support the conclusions above. The low P4 values obtained in isolated mitochondria when StAR is present after CCCP wash-out mimic results observed in whole MA-10 cells. The presence of both active kinases promotes a significant increase in P4 production, thereby validating the obligatory role of ERK and PKA as required mediators in mitochondria to elicit the maximal steroidogenesis rate. These conclusions are supported by the lack of effect of ERK and PKA inhibitors on cellular P4 production in Phase II. If the accesibility of PKA and ERK to the mitochondria is allowed when mitochondrial StAR is present, then P4 production is maximal.

In this work we have clearly shown that mitochondrial fusion through the increase of Mfn2 levels in mitochondria is strictly required for StAR protein synthesis and mitochondrial localization after hormone stimulation by hCG and cAMP. Mitochondrial fusion reduction correlates with a decrease in StAR protein in mitochondria and mRNA levels. The results obtained with Mfn2 knockdown are entirely consistent with those observed with CCCP treatments, suggesting that the effect of CCCP might be partially due to mitochondrial fusion inhibition. In this regard, we have demonstrated here that ERK mitochondrial activity depends on Mfn2 presence in mitochondria, in agreement with the lack of steroidogenic capacity of cells in the presence of CCCP and with previous published work [33].

A direct role of Mfn2 on StAR gene expression is unknown to date. In this regard, a previous report shows that over-expression or knockout of Mfn2 modulates and affects mRNA levels of several mitochondrial proteins [51], [52]. We have described an arachidonic acid [55] generation/exportation system that includes an Acyl-CoA synthetase 4 (Acsl4) [38], [40], [56]. Acsl4 is anchored at the MAM (mitochondrial associated-membrane) structures [57] and its activity determines the production rate of AA which is necessary for StAR gene expression [22]. Our previous work demonstrated that mitochondrial fusion to the ER (MAM) is required for the correct localization of Acsl4, after hormone stimulation [33]. Therefore, mitochondrial fusion can be suggested to participate in StAR synthesis regulation through Acsl4 regulation and MAMs localization; thereby increasing the intramitochondrial AA and leukotrienes production. It is worth mentioning that a stabilizing effect of mitochondrial fusion on StAR mRNA cannot be excluded. Interestingly, a report has proposed that the different variants of StAR mRNA levels are regulated by stabilization mediated by cAMP in MA-10 cells [41], [49]. Recently, StAR mRNA was shown to bind a mitochondrial AKAP1 stabilizing the translational complex at this organelle [50]. Therefore, mitochondrial fusion could mediate the approach between StAR mRNA and the AKAP1 in mitochondria, thus stabilizing and increasing StAR mRNA levels, as shown in this work.

**Figure 8:**
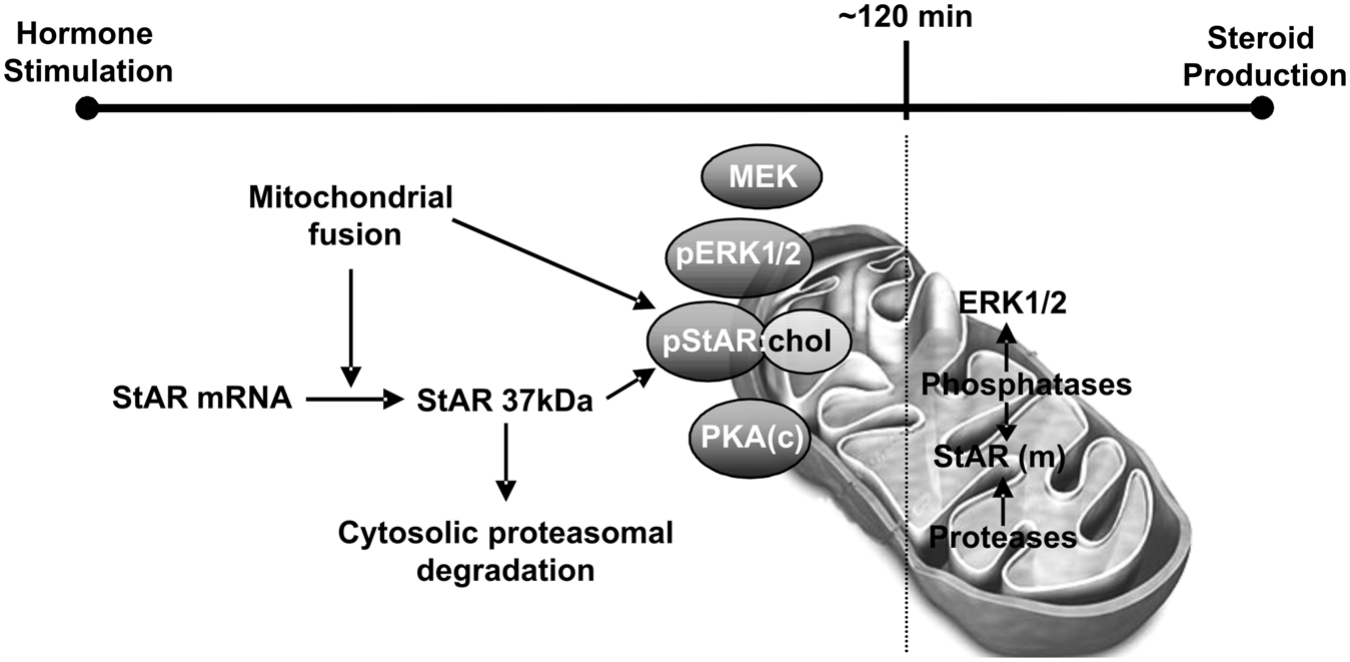
Summary of the proposed mechanisms for the association of StAR to the OMM in MA-10 Leydig cells following hormone stimulation. After hormone stimulation, mitochondrial fusion induction through Mfn2 up-regulation in mitochondria is needed for increasing StAR mRNA levels. Also, mitochondrial fusion is required post-transcriptionally for StAR localization at the OMM and subsequent binding to a cholesterol molecule. Then, cholesterol-bound StAR is available for ERK and PKA mitochondrial phosphorylation, increasing its activity and cholesterol transport. After the 2 h temporal frame of ERK activity, StAR is dephosphorylated, de-activated and translocated to the mitochondrial matrix where it is degraded by mitochondrial proteases. Abbreviations: pERK1/2 (phospho-ERK1/2), pStAR: chol (phospho-StAR bound to a cholesterol molecule), PKA(c) (PKA catalytic subunit), StAR (m) (StAR in the mitochondrial matrix).

Figure 8 depicts mechanisms involved in StAR activity associated with the transduceome localized on the OMM, including the relevance of mitochondrial fusion and ERK activity on StAR as fundamental steps. This representation is fully consistent with the mechanism proposed by Miller and co-workers [12], [14].

## Conclusions

Briefly, we may conclude that mitochondrial fusion stimulated by steroidogenic hormones, through the cAMP pathway, defines not only StAR mRNA levels in the cell but also the localization of active StAR protein in the mitochondria. Indeed, the StAR phospho/dephosphorylation cycle could be mediated by ERK activity and mitochondrial phosphatases in the context of the OMM, enabling a very small amount of mitochondrial StAR to be responsible for the very large number of steroid molecules produced in a short period of time.

## Supporting Information

Figure S1 **Effect of CCCP on StAR mRNA levels.** Cells were subjected to the two-phase experiment. Total RNA was isolated, reverse transcribed and subjected to semi-quantitative PCR using specific primers for StAR and L19 cDNA, as loading control. PCR products were resolved in ethidium bromide-stained agarose gels. Treatments and corresponding phases are indicated in the figure. A representative gel is shown. For each band, the OD of the expression levels of StAR were quantified (arbitrary units) and normalized to the corresponding L19 abundance. The relative levels of StAR are shown: **p<0.001 vs. Phase I; ¥¥¥ p<0.001 vs. control; ns p>0.05 vs. control CCCP. All the results are expressed as means ± SEM of three independent experiments.
(TIF)

Table S1 **Effect of CCCP on P4 production sustained by 22(R)-OH-cholesterol.** P4 production was measured by RIA in the culture media in the presence or absence of 22(R)-OH-cholesterol and CCCP, at the indicated times. Data are shown as P4 concentration (ng/ml). *ns p>0.05 without CCCP vs. with CCCP.
(TIF)
