# Supplementary figures and images for: Mitochondrial Fusion and ERK Activity Regulate Steroidogenic Acute Regulatory Protein Localization in Mitochondria
Source: PLoS One. 2014 Jun 19;9(6):e100387. doi: 10.1371/journal.pone.0100387 (PMC4063759; doi:10.1371/journal.pone.0100387)

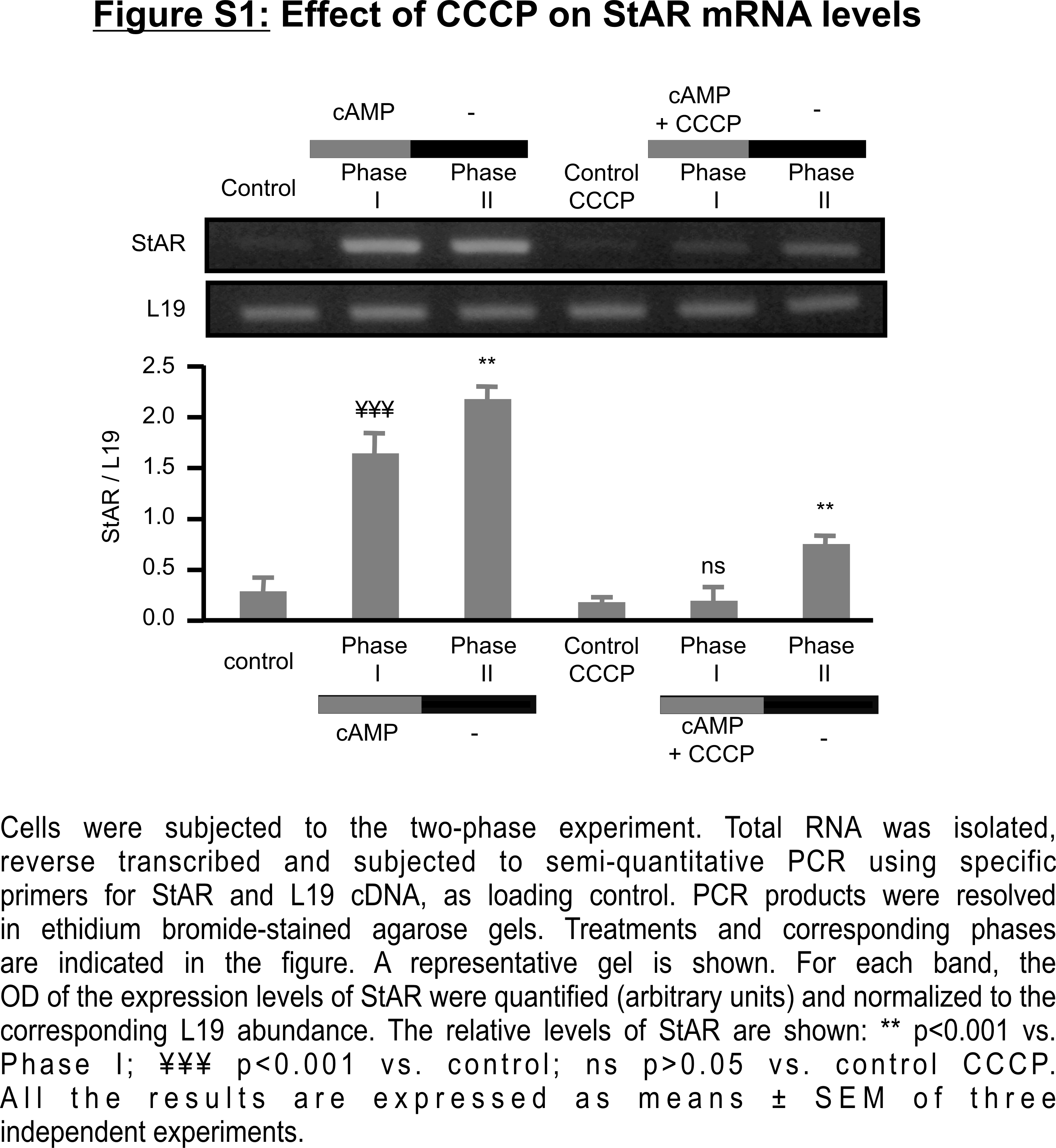

Supplement: Figure S1 — Effect of CCCP on StAR mRNA levels. Cells were subjected to the two-phase experiment. Total RNA was isolated, reverse transcribed and subjected to semi-quantitative PCR using specific primers for StAR and L19 cDNA, as loading control. PCR products were resolved in ethidium bromide-stained agarose gels. Treatments and corresponding phases are indicated in the figure. A representative gel is shown. For each band, the OD of the expression levels of StAR were quantified (arbitrary units) and normalized to the corresponding L19 abundance. The relative levels of StAR are shown: **p<0.001 vs. Phase I; ¥¥¥ p<0.001 vs. control; ns p>0.05 vs. control CCCP. All the results are expressed as means ± SEM of three independent experiments. (TIF) [file pone.0100387.s001.tif]

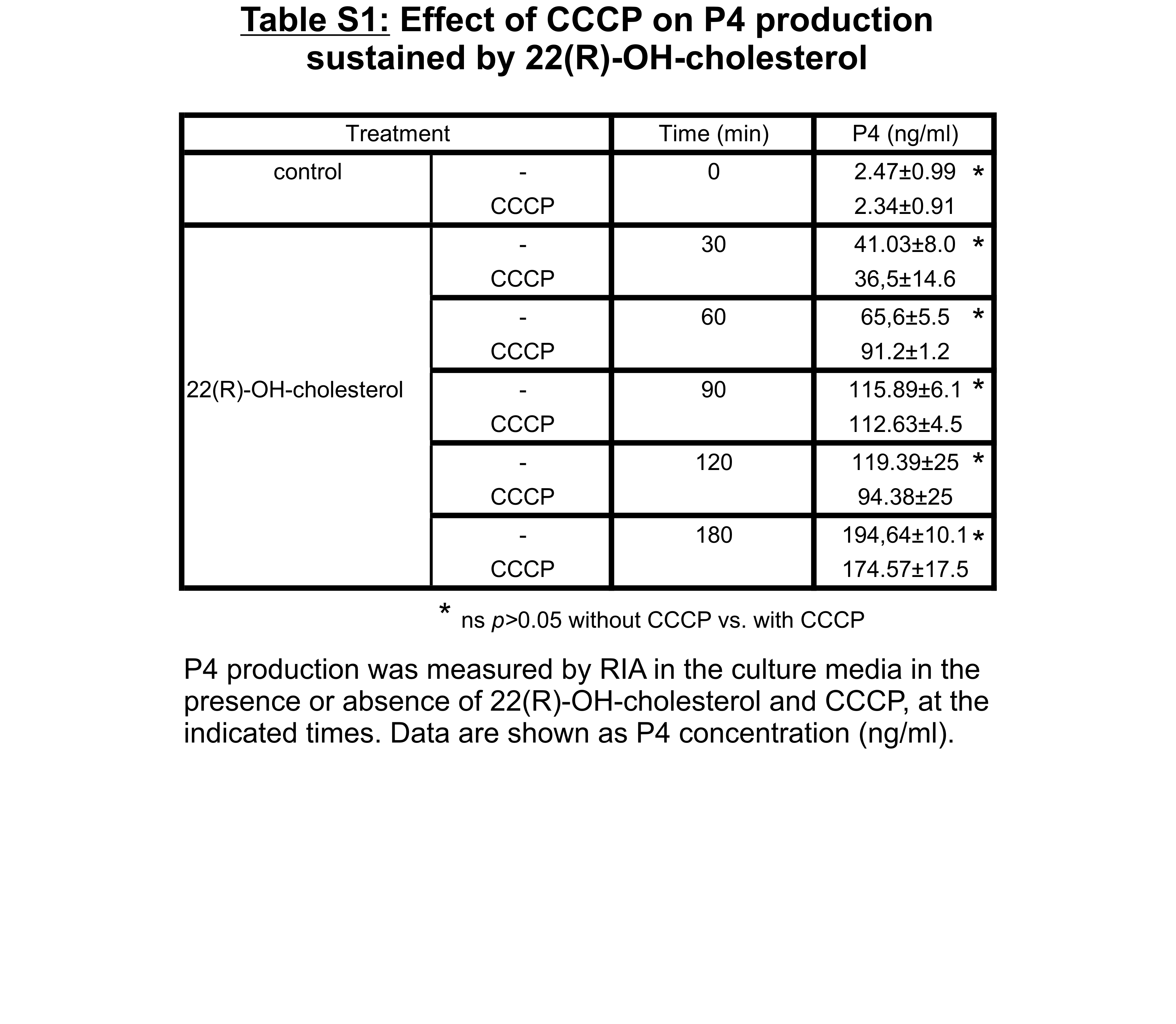

Supplement: Table S1 — Effect of CCCP on P4 production sustained by 22(R)-OH-cholesterol. P4 production was measured by RIA in the culture media in the presence or absence of 22(R)-OH-cholesterol and CCCP, at the indicated times. Data are shown as P4 concentration (ng/ml). *ns p>0.05 without CCCP vs. with CCCP. (TIF) [file pone.0100387.s002.tif]
